# A comparative examination of the role of need in the relationship between dental service use and socio-economic status across respondents with distinct needs using data from the Scottish Health Survey

**DOI:** 10.1186/s12889-023-15078-z

**Published:** 2023-01-24

**Authors:** Majed Almutairi, Gerry McKenna, Ciaran O’Neill

**Affiliations:** 1 Centre for Public Health, School of Medicine, Dentistry and Biomedical Sciences, Queen’s University Belfast, Belfast, UK; 2 Public Health Department, School of Health Sciences, Saudi Electronic University, Riyadh, Saudi Arabia

**Keywords:** Access to care, Disparities, Dental services, Utilization, Inequalities

## Abstract

**Background:**

Disparities in oral health and distinct patterns in service use related to socio-economic status have been shown to exist in the United Kingdom. A number of studies have used the Andersen behavioural model to better understand the factors that influence utilization and thereby inform policies aimed at improving service uptake. As the nature of need may differ across distinct types of patients, however, so too may the distribution of enabling and pre-disposing factors and observed relationships between need, other factors and service use. In this study we compare samples with distinct self-assessed needs in terms of their characteristics and patterns of service use to compare application of the Andersen model to dental services among respondents to a population based survey.

**Materials and methods:**

Data were taken from the Scottish Health Survey, for 2019. Data on service use, oral hygiene habits, perceived treatment need, and socio-demographic characteristics were extracted. Data were analysed using descriptive statistics, t-tests and ordered logistic regression analyses.

**Results:**

Two thousand one hundred forty-eight usable responses were obtained from the survey, 74.95% of the sample had visited the dentist less than a year ago, 11.82% between 1 year and up to 2 years ago, 7.12% between 2 and 5 years ago and 6.10% more than 5 years. Descriptive statistics, t-tests and ordered logistic regression analyses revealed distinct patterns of service use when the sample was partitioned based on perceived treatment need. Specifically those with self-assessed treatment need were older, more likely to smoke, be male and be less likely to have a degree than those who did not. While service use was positively related to age (predisposing) among those who did not have self-assessed treatment need, it was negatively related for those with perceived treatment need. Distinct patterns were also evident with respect to sugar exposure (need) and ease with which time off work could be organised (enabling).

**Discussion:**

The study shows common and distinct patterns of service use related to enabling and predisposing factors across groups differentiated by self-perceived treatment need. If inequalities in health and healthcare use are to be addressed, it is important to understand their origins. Conflation of distinct types of need that may correlate with predisposing and enabling factors complicates this.

**Conclusion:**

In applying the Andersen model, it is important to take account of potential differences in the types of need expressed where possible to understand the role of other variables in service use.

**Supplementary Information:**

The online version contains supplementary material available at 10.1186/s12889-023-15078-z.

## Background

Disparities in oral health related to socio-economic status (SES) have been presented in the literature for some time [1–6]. With respect to both subjective and objective measures of, those with lower SES have been found to have poorer oral health [7–12]. Studies have suggested that this is grounded in a range of factors that include access to material resources [13], self-esteem [14], cognitive ability [15] and health literacy [16] all of which may directly or indirectly through access to service impact of health. While oral health has improved in the UK over time, inequalities persist [1, 17–19] and dental services can play an important role in health improvement and the reduction of oral health inequalities [20]. Inequalities related to SES in use of care, however, have been evident including in the UK for some time [21]. While improving access to services is acknowledged as central to efforts to improve health and reduce inequalities [22], unless we understand the factors that unpin differential uptake of services it will be challenging to optimally devise policies that serve to address the factors underpinning inequalities in use and health.

Different models have been proposed to try and better understand the factors that influence healthcare utilization. The Andersen Behavioural Model is one that has been used extensively in the literature [23, 24], including with respect to dental services [25]. In brief, the model seeks to explain service use by reference to a range of observable characteristics possessed by the potential user. To illuminate the reasons underlying use, variables are grouped under three headings: predisposing, enabling and need factors. Pre-disposing factors are those make a person more likely to use services such as age, education and cultural norms. These may result in a person being more aware of the existence or benefits of services, for example. Enabling factors include those related to the affordability of services including the burden charges may present or that eligibility for support may offer; barriers to service access related to waiting times, travel times or challenges in obtaining time off work to use services. Need, relates to both perceived and objectively measured need covering, for example, perceived treatment need as well as pain and with specific regard to oral health the number of decayed or missing teeth. Such factors may directly influence the perceived benefits of service use or the impact on quality of life in terms of function, pain or aesthetics, of non-use. Collectively they may help guide policy in efforts to address inequalities in service use and consequently health by identifying specific barriers that allow targeted intervention for particular groups such as changes in employment rights, public funding or tailored health promotion.

A recent systematic review of the literature applying the Andersen Model to dental services found evidence of a consistent role for pre-disposing factors (such as age), enabling factors (such as income) and need factors (such as measures of oral health) in explaining differential use among children [26]. Less consistent evidence as to the role accorded these factors was evident with respect to adults though about one half of the studies reviewed did find evidence of a positive relationship between education and dental service use. In studies specific to the UK, perceived treatment need and the number of decayed or missing teeth have been shown to influence service use as has difficulty in accessing services (enabling factors) and expense (enabling), as have predisposing factors such as education [21, 25]. The popularity of the Andersen Model is evident as is its potential in principle to support policy development.

Challenges exist with the application of the model in practice, however, that may help explain the equivocal results obtained when applying the model to adults. Dental care is delivered predominately by general dental practitioners who are generally self-employed for-profit providers. Across jurisdictions, different funding arrangements exist providing varying degrees of support in access to care for adults. In the UK for example, financial support is available to access care but varies depending on the age and income of the person concerned [27]. Care is therefore warranted in assessing the role of predisposing factors such as age given it may also effect enabling factors related to financial barriers and create issues of endogeneity when estimating relationships. Some have sought to address this through structural equation modelling which allows for more complex relationships than “simple” regression analysis including indirect pathways through which enabling factors may impact need and subsequently service use and health. However, these remain problematic. General dental practitioners provide a range of services that include treatment and prevention. While those who use services may be able to avail of [20, 21] all services, for example, in reality distinct patterns of service use may exist between different types of patient [28, 29]. Some who are regular attenders may have relatively speaking good oral health and be more likely to consume preventive services, for example. By contrast, others who are irregular attenders may exhibit distinct patterns of service use in which restoration and extraction feature more prominently related to poorer oral health and acute problems. Conflating distinct types of patient within an analysis may effectively conflate distinct types of need and contribute to the equivocal results reported in the literature in terms of the role of variables in use among adults.

In this paper, we apply the Andersen in a pooled sample of service users before repeating the analysis among service users differentiated on the basis of their self-assessed treatment needs to ascertain if models estimated for sub-groups differentiated by need provide additional insights with respect to service use.

## Materials

Data were taken from the Scottish Health Survey, a cross sectional representative survey of adults in Scotland for 2019. The data comprised socio-demographic characteristics of the respondent including age, sex, education, income, and smoking status. Oral hygiene habits covered tooth brushing, flossing, use of mouth rinse and behaviours related to sugary drinks. Respondents also reported self-assessed treatment need as well as how recently dental services had been used (specified in the survey as less than year, more than one year- up to two years, more than two years up to five years, more than five years and never). Analyses were restricted to those for whom all data were available; there was no attempt to impute missing values.

## Methods

To test the effect of partitioning the sample the analyses were repeated for the full sample and when the sample was partitioned based on whether the respondent expressed a treatment need or not. The decision to partition the sample was taken prior to analyses. The analyses comprised descriptive statistics, t-test for differences between those with perceived treatment need and those without and an ordered logit to take account of the ordinal nature of the utilization data. Ordered logistic regression analyses were used to reflect the ordinal nature of the dependent variable which captured how recently the respondent had visited the dentist.The choice of variables used in regression analysis was informed by the literature [21, 26]. Details of how each variable was specified are set out in Appendix 1. Original ethical approval for the Scottish Health Survey 2019 was granted by the Research Committee for Wales (17/WA/0371) and participants gave full informed consent to participate in the study. Anonymised data are accessible via the UK Data Archive for which no additional ethical approval was required.

## Results

Table 1 sets out descriptive statistics for the entire sample and Tables 2 and 3 those for the sample who reported perceived treatment need and those who did not report perceived treatment need respectively. As can be seen distinct patterns are evident in the sample characteristics when partitioned on the basis of perceived treatment need. For example, while among the combined sample approximately 34% had a degree or above as their highest qualification among those with perceived need the figure was approximately 14 percentage points lower than among those with no perceived treatment need. Similarly, those with perceived need were more likely to smoke, more likely to be male, less likely to be over 65, more likely to encounter issues with getting time off work, finding suitable appointment times, issues with dental expenses and were less likely to floss or avoid sugar in their diets. As seen in Table 4 those who with perceived need also exhibit distinct visitation patterns to those without perceived needs, a higher percentage of those without perceived need visiting within the past year, for example, and a lower percentage visiting at less recently. Statistical differences in respect of specific variables are highlighted in Table 5 and underscore the differences between the two groups with respect to visits and a range of characteristics that may predispose or enable them to visit the dentist. Those with perceived treatment need for example are more likely to smoke (while for example, 23% of those without treatment need smoked, 38% of those with treatment need smoked, t = -7.47, *p* < 0.001), less likely to have a degree (38% of those without treatment need hade a degree, 24% of those with treatment need had a degree, t = 6.66, *p* < 0.001), less likely to be over 65 ( 25% of those without treatment need were over 65, 19% of those with treatment need were over 65%, t = 3.13, *p* < 0.05) and less likely to be female ( 46% of those without treatment need were male, 53% of those with treatment need were male, t = 3.12, *p* < 0.05). By contrast those with no perceived treatment need are more likely to brush (97% of those without treatment need brushed their teeth, 93% with treatment need is brushed their teeth, t = 4.52, *p* < 0.001), floss ( 35% of those without treatment need used floss, 25% with treatment need used floss, t = 4.75, *p* < 0.001) and avoid sugar ( 31% of those without treatment need avoided sugar, 22% with treatment needed avoid sugar, t = 4.21, *p* < 0.001), while there are no differences in terms of the issues with distance to the dentist between groups.

**Table 1:** Descriptive statistics for the entire sample

Variable	Mean	Std. Err	[95% Conf. Interval]	
**Have a degree**	**.3384544**	**.0102121**	**.3184278**	**.358481**
**Male**	**.4846369**	**.0107857**	**.4634853**	**.5057884**
**Over 65 years old**	**.2369646**	**.0091769**	**.218968**	**.2549612**
**Equivalised household income quintiles**				
** 1**	**.1755121**	**.0082097**	**.1594122**	**.191612**
** 2**	**.198324**	**.0086054**	**.1814482**	**.2151998**
** 3**	**.1978585**	**.0085978**	**.1809976**	**.2147193**
** 4**	**.2295158**	**.0090755**	**.2117181**	**.2473136**
** 5**	**.1987896**	**.008613**	**.1818989**	**.2156802**
**Smoker**	**.2853818**	**.0097462**	**.2662688**	**.3044947**
**perceived dental need**	**.3361266**	**.0101948**	**.3161339**	**.3561193**
**Difficulty in getting time off work**	**.0577281**	**.0050335**	**.0478572**	**.0675991**
**Difficulty in getting an appointment at suitable time**	**.0758845**	**.0057151**	**.0646768**	**.0870923**
**Dental treatment is too expensive**	**.066108**	**.0053624**	**.055592**	**.076624**
**Long way to see a dentist**	**.0446927**	**.0044594**	**.0359476**	**.0534379**
**Can't find dentist liked**	**.023743**	**.0032857**	**.0172994**	**.0301866**
**Can't get NHS care**	**.0218808**	**.0031573**	**.0156892**	**.0280724**
**Difficulty accessing building**	**.0069832**	**.0017972**	**.0034589**	**.0105076**
**Other problems**	**.0069832**	**.0017972**	**.0034589**	**.0105076**
**Brush their teeth**	**.9581006**	**.0043241**	**.9496207**	**.9665804**
**Use of floss**	**.3175047**	**.0100464**	**.297803**	**.3372063**
**Use of mouth rinse**	**.3831471**	**.010492**	**.3625716**	**.4037226**
**Restrict their intake of sugary foods and drinks**	**.2816574**	**.0097076**	**.2626201**	**.3006946**

*N* = 2,148

Equivalised household income quintiles were reported in the survey, where 1 is the lowest and 5 the highest quintile

**Table 2:** Descriptive statistics for those who reported perceived treatment need

Variable	Mean	Std. Err	[95% Conf. Interval]	
**Degree**	**.2437673**	**.01599**	**.2123748**	**.2751598**
**Male**	**.531856**	**.0185831**	**.4953724**	**.5683395**
**Over65**	**.1966759**	**.0148031**	**.1676135**	**.2257383**
**Equivalised household income quintiles**				
** 1**	**.2216066**	**.0154676**	**.1912397**	**.2519736**
** 2**	**.2271468**	**.0156039**	**.1965122**	**.2577814**
** 3**	**.1925208**	**.0146837**	**.1636928**	**.2213488**
** 4**	**.2049861**	**.0150343**	**.17547**	**.2345023**
** 5**	**.1537396**	**.0134331**	**.1273669**	**.1801123**
**Smoker**	**.3864266**	**.0181342**	**.3508244**	**.4220288**
**Difficulty in getting time off work**	**.0803324**	**.0101226**	**.0604591**	**.1002058**
**Difficulty in getting an appointment at suitable time**	**.1191136**	**.0120635**	**.0954298**	**.1427973**
**Dental treatment is too expensive**	**.1218837**	**.0121838**	**.0979638**	**.1458035**
**Long way to see a dentist**	**.0498615**	**.008106**	**.0339472**	**.0657757**
**Can't find dentist liked**	**.0567867**	**.0086191**	**.0398652**	**.0737082**
**Can't get NHS care**	**.0415512**	**.0074321**	**.0269602**	**.0561423**
**Difficulty accessing building**	**.0069252**	**.0030884**	**.0008618**	**.0129886**
**Other problems**	**.0069252**	**.0030884**	**.0008618**	**.0129886**
**Brush their teeth**	**.9307479**	**.0094551**	**.9121852**	**.9493107**
**Use of floss**	**.2506925**	**.0161411**	**.2190034**	**.2823817**
**Use of mouth rinse**	**.4099723**	**.0183166**	**.374012**	**.4459326**
**Restrict their intake of sugary foods and drinks**	**.2243767**	**.0155363**	**.193875**	**.2548785**

*N *= 722

**Table 3:** Descriptive statistics for those who did not report perceived treatment need

Variables	Mean	Std. Err	[95% Conf. Interval]	
**How recently a dentist was visited**	**1.21669**	**.0165717**	**1.184183**	**1.249198**
**Degree**	**.3863955**	**.0128989**	**.3610926**	**.4116984**
**Male**	**.4607293**	**.0132044**	**.4348272**	**.4866315**
**Over65**	**.2573633**	**.0115812**	**.2346452**	**.2800813**
**Equivalised household income quintiles**				
** 1**	**.1521739**	**.0095152**	**.1335087**	**.1708391**
** 2**	**.1837307**	**.0102589**	**.1636066**	**.2038549**
** 3**	**.200561**	**.0106074**	**.1797532**	**.2213688**
** 4**	**.2419355**	**.0113448**	**.2196813**	**.2641897**
** 5**	**.2215989**	**.0110022**	**.2000167**	**.2431811**
**Smoker**	**.2342216**	**.0112191**	**.2122139**	**.2562293**
**Difficulty in getting time off work**	**.0462833**	**.0055656**	**.0353656**	**.057201**
**Difficulty in getting an appointment at suitable time**	**.0539972**	**.0059872**	**.0422525**	**.0657419**
**Dental treatment is too expensive**	**.0378682**	**.0050565**	**.0279492**	**.0477871**
**Long way to see a dentist**	**.0420757**	**.0053183**	**.0316432**	**.0525083**
**Can't find dentist liked**	**.0070126**	**.0022106**	**.0026763**	**.0113489**
**Can't get NHS care**	**.0119215**	**.0028751**	**.0062816**	**.0175613**
**Difficulty accessing building**	**.0070126**	**.0022106**	**.0026763**	**.0113489**
**Other problems**	**.0070126**	**.0022106**	**.0026763**	**.0113489**
**Brush their teeth**	**.9719495**	**.0043741**	**.9633692**	**.9805298**
**Use of floss**	**.3513324**	**.0126463**	**.3265251**	**.3761397**
**Use of mouth rinse**	**.3695652**	**.0127867**	**.3444825**	**.394648**
**Restrict their intake of sugary foods and drinks**	**.3106592**	**.0122589**	**.2866118**	**.3347066**

*N *= 1426

**Table 4:** Descriptive statistics for sample partitioned by perceived treatment need

**Perceived treatment need = No**			
**How recently a dentist was visited**	Freq	Percent	Cum
**Less than a year ago**	1,238	86.82	86.82
**More than 1 year, up to 2 years ago**	104	7.29	94.11
**More than 2 years, up to 5 years ago**	47	3.30	97.41
**More than 5 years ago**	37	2.59	100.00
** Total**	1,426	100.00	
**Perceived treatment need = Yes**			
**How recently a dentist was visited**	Freq	Percent	Cum
**Less than a year ago**	372	51.52	51.52
**More than 1 year, up to 2 years ago**	150	20.78	72.30
**More than 2 years, up to 5 years ago**	106	14.68	86.98
**More than 5 years ago**	94	13.02	100.00
** Total**	722	100.00	
**Full sample perceived treatment need = Yes or No**			
**How recently a dentist was visited**	Freq	Percent	Cum
**Less than a year ago**	1,610	74.95	74.95
**More than 1 year, up to 2 years ago**	254	11.82	86.78
**More than 2 years, up to 5 years ago**	153	7.12	93.90
**More than 5 years ago**	131	6.10	100.00
** Total**	2,148	100.00

**Table 5:**
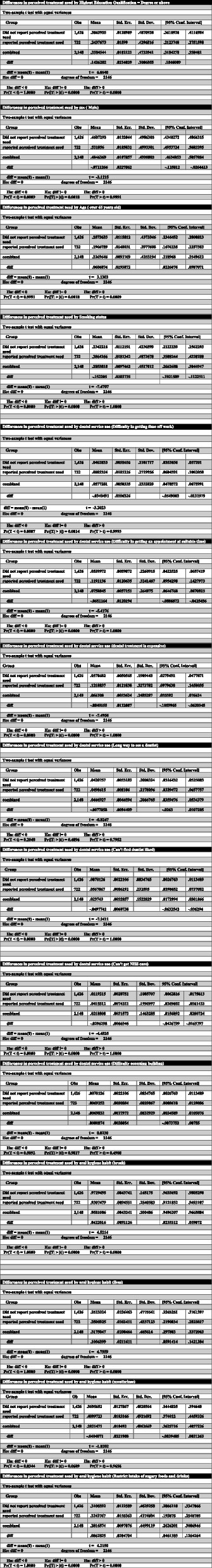
T-tests of enabling and predisposing factors by perceived treatment need

In Tables 6, 7 and 8 the results of ordered logistic regressions that examine the relationship between how recently a dentist was visited and the range of predisposing, enabling and need factors are shown for the combined, perceived treatment necessary and no perceived treatment necessary groups respectively are shown. The results demonstrate distinct patterns in relationships between the two sub-groups that are masked when they are combined. For example, with respect to age – a predisposing factor—in the combined sample reported in Table 6 no significant relationship with how recently the dentist was visited is found (z = -0.1, *p* = 0.92). This is similarly the case with respect to time off work – an enabling factor – and efforts to avoid sugar that could be construed as a need factor. When examining the two groups separately, however, we see in Table 7 that for those with perceived treatment need, age is significantly related to having visited the dentist less recently while among those with no perceived treatment need the reverse is true, (Table 8) being associated with more recent visits. Similarly, with respect to time off work while this is an issue for those with no perceived need being significantly associated with less recent visits, it is not significant for those with perceived treatment needs, whereas avoidance of sugar is related to more recent visits among this group but unrelated for those without perceived treatment needs.

**Table 6:** Ordered logistic regression of service use as function of need, predisposing and enabling variables for entire sample

How recently a dentist was visited	Coef	Std. Err	z	P >|z|	[95% Conf. Interval]	
**Have a degree**	**-.0099061**	**.1237052**	**-0.08**	**0.936**	**-.2523638**	**.2325517**
**Male**	**.2542373**	**.112279**	**2.26**	**0.024**	**.0341745**	**.4743002**
**Over 65 years old**	**-.0139261**	**.1443814**	**-0.10**	**0.923**	**-.2969085**	**.2690564**
**Equivalised household income quintiles**						
** 2**	**.2997523**	**.1744271**	**1.72**	**0.086**	**-.0421186**	**.6416232**
** 3**	**.0253468**	**.1812751**	**0.14**	**0.889**	**-.3299458**	**.3806394**
** 4**	**.0089851**	**.1728139**	**0.05**	**0.959**	**-.3297239**	**.3476942**
** 5**	**-.1034873**	**.1922235**	**-0.54**	**0.590**	**-.4802385**	**.273264**
**Smoker**	**.3169925**	**.1212232**	**2.61**	**0.009**	**.0793994**	**.5545855**
**perceived dental need**	**1.636239**	**.1186513**	**13.79**	**0.000**	**1.403687**	**1.868791**
**Difficulty in getting time off work**	**.4214942**	**.2344101**	**1.80**	**0.072**	**-.037941**	**.8809295**
**Difficulty in getting an appointment at suitable time**	**-.192323**	**.1964428**	**-0.98**	**0.328**	**-.5773438**	**.1926978**
**Dental treatment is too expensive**	**.1256905**	**.1828419**	**0.69**	**0.492**	**-.232673**	**.4840541**
**Long way to see a dentist**	**-.6699822**	**.3201704**	**-2.09**	**0.036**	**-1.297505**	**-.0424597**
**Can't find dentist liked**	**.779781**	**.2796835**	**2.79**	**0.005**	**.2316115**	**1.327951**
**Can't get NHS care**	**.4272969**	**.2830344**	**1.51**	**0.131**	**-.1274403**	**.9820341**
**Difficulty accessing building**	**.178959**	**.5524285**	**0.32**	**0.746**	**-.9037809**	**1.261699**
**Brush their teeth**	**-1.043539**	**.2501673**	**-4.17**	**0.000**	**-1.533858**	**-.5532201**
**Use of floss**	**-.9570342**	**.1375998**	**-6.96**	**0.000**	**-1.226725**	**-.6873435**
**Use of mouth rinse**	**-.2270864**	**.1121358**	**-2.03**	**0.043**	**-.4468686**	**-.0073042**
**Restrict their intake of sugary foods and drinks**	**-.1986253**	**.1360909**	**-1.46**	**0.144**	**-.4653586**	**.0681079**
** /cut1**	**.7382189 .3015972**				**.1470992**	**1.329339**
** /cut2**	**1.706602 .3023276**				**1.114051**	**2.299154**
** /cut3**	**2.684965 .3088323**				**2.079665**	**3.290265**

Wald chi2(20) = 402.43, *N* = 2,148

Income quintile 1 is the base category for income

**Table 7:** Ordered logistic regression of service use as function of need, predisposing and enabling variables for those who reported perceived treatment need

How recently a dentist was visited	Coef	Std. Err	z	P >|z|	[95% Conf. Interval]	
**Have a degree**	**.0688728**	**.1810842**	**0.38**	**0.704**	**-.2860458**	**.4237914**
**Male**	**.3312819**	**.1532129**	**2.16**	**0.031**	**.0309901**	**.6315737**
**Over 65 years old**	**.4413698**	**.2162408**	**2.04**	**0.041**	**.0175456**	**.865194**
**Equivalised household income quintiles**						
** 2**	**.6366216**	**.2267426**	**2.81**	**0.005**	**.1922142**	**1.081029**
** 3**	**.2583604**	**.2360571**	**1.09**	**0.274**	**-.2043029**	**.7210237**
** 4**	**.3209828**	**.2166287**	**1.48**	**0.138**	**-.1036017**	**.7455673**
** 5**	**.368294**	**.2569402**	**1.43**	**0.152**	**-.1352994**	**.8718875**
**Smoker**	**.1809394**	**.1545142**	**1.17**	**0.242**	**-.1219028**	**.4837815**
**Difficulty in getting time off work**	**.0093941**	**.309416**	**0.03**	**0.976**	**-.5970502**	**.6158384**
**Difficulty in getting an appointment at suitable time**	**-.3324231**	**.232886**	**-1.43**	**0.153**	**-.7888712**	**.124025**
**Dental treatment is too expensive**	**.0737112**	**.2087965**	**0.35**	**0.724**	**-.3355224**	**.4829448**
**Long way to see a dentist**	**-.9137185**	**.4138208**	**-2.21**	**0.027**	**-1.724792**	**-.1026446**
**Can't find dentist liked**	**.848322**	**.2882627**	**2.94**	**0.003**	**.2833375**	**1.413307**
**Can't get NHS care**	**.250926**	**.3633885**	**0.69**	**0.490**	**-.4613023**	**.9631544**
**Difficulty accessing building**	**.4678508**	**.9019082**	**0.52**	**0.604**	**-1.299857**	**2.235558**
**Brush their teeth**	**-.7316026**	**.2766107**	**-2.64**	**0.008**	**-1.27375**	**-.1894555**
**Use of floss**	**-1.072839**	**.187329**	**-5.73**	**0.000**	**-1.439998**	**-.7056813**
**Use of mouth rinse**	**-.1645115**	**.1476945**	**-1.11**	**0.265**	**-.4539874**	**.1249644**
**Restrict their intake of sugary foods and drinks**	**-.4466689**	**.2073113**	**-2.15**	**0.031**	**-.8529917**	**-.0403462**
** /cut1**	**-.3967924**	**.3209894**			**-1.02592**	**.2323352**
** /cut2**	**.6077337**	**.3211487**			**-.0217062**	**1.237174**
** /cut3**	**1.635568**	**.3260823**			**.9964586**	**2.274678**

Wald chi2(19) = 84.66, *N* = 722

Income quintile 1 is the base category for income

**Table 8:** Ordered logistic regression of service use as function of need, predisposing and enabling variables for those who did not report perceived treatment need

How recently a dentist was visited	Coef	Std. Err	z	P >|z|	[95% Conf. Interval]	
**Have a degree**	**-.0326908**	**.1815993**	**-0.18**	**0.857**	**-.3886189**	**.3232374**
**Male**	**.2709913**	**.1695045**	**1.60**	**0.110**	**-.0612313**	**.6032139**
**Over 65 years old**	**-.5242293**	**.2334142**	**-2.25**	**0.025**	**-.9817127**	**-.0667459**
**Equivalised household income quintiles**						
** 2**	**-.212868**	**.2675791**	**-0.80**	**0.426**	**-.7373135**	**.3115774**
** 3**	**-.4230927**	**.2703426**	**-1.57**	**0.118**	**-.9529545**	**.106769**
** 4**	**-.5177904**	**.2744095**	**-1.89**	**0.059**	**-1.055623**	**.0200423**
** 5**	**-.7030163**	**.2925729**	**-2.40**	**0.016**	**-1.276449**	**-.129584**
**Smoker**	**.5021803**	**.1927514**	**2.61**	**0.009**	**.1243946**	**.8799661**
**Difficulty in getting time off work**	**.8537863**	**.3083808**	**2.77**	**0.006**	**.249371**	**1.458202**
**Difficulty in getting an appointment at suitable time**	**.0995622**	**.3004786**	**0.33**	**0.740**	**-.4893651**	**.6884895**
**Dental treatment is too expensive**	**.2678468**	**.3635152**	**0.74**	**0.461**	**-.44463**	**.9803236**
**Long way to see a dentist**	**-.4014025**	**.4141612**	**-0.97**	**0.332**	**-1.213144**	**.4103385**
**Can't find dentist liked**	**1.095444**	**.8695461**	**1.26**	**0.208**	**-.6088349**	**2.799723**
**Can't get NHS care**	**.5245527**	**.4118075**	**1.27**	**0.203**	**-.2825752**	**1.331681**
**Difficulty accessing building**	**-.2886625**	**.9142258**	**-0.32**	**0.752**	**-2.080512**	**1.503187**
**Brush their teeth**	**-1.894596**	**.4301708**	**-4.40**	**0.000**	**-2.737715**	**-1.051477**
**Use of floss**	**-.8467604**	**.2057667**	**-4.12**	**0.000**	**-1.250056**	**-.4434651**
**Use of mouth rinse**	**-.28335**	**.1793761**	**-1.58**	**0.114**	**-.6349207**	**.0682206**
**Restrict their intake of sugary foods and drinks**	**.0112407**	**.182455**	**0.06**	**0.951**	**-.3463645**	**.3688459**
** /cut1**	**-.4207167**	**.4898347**			**-1.380775**	**.5393416**
** /cut2**	**.5275815**	**.487147**			**-.4272091**	**1.482372**
** /cut3**	**1.428826**	**.5021521**			**.4446263**	**2.413026**

Wald chi2(19) = 98.56, *N* = 1426

Income quintile 1 is the base category for income

## Discussion

Inequalities in oral health and healthcare exist in many jurisdictions. They have been shown to persist in the UK despite various efforts to improve access to preventive and treatment services as well as broader public health initiatives that might impact on need [29]. While the Andersen model has been used to inform modelling studies that seek to explain variations in service utilization (and subsequently health) these have not always differentiate between the distinct types of need those potential users may exhibit [21]. A failure to examine separately preventive and restorative (or other treatments for established disease) care needs, given the probability that these have distinct relationships with the socio-demographic characteristics of those who express them, raises the possibility of utilization models being mis-specified and erroneous inferences being drawn as a result. It may also in part explain the inconsistent relationships for adults reported in the literature found using the Andersen model [26].

Our analysis clearly shows the existence of distinct sample sub-groups based on perceived treatment need. Those who self-assess perceived treatment needs are older, less well educated, more likely to be male, more likely to smoke and less likely to have good oral hygiene habits than those who do not express such needs. Such factors likely interact to influence oral health and use of oral health services rather than simply be associated with distinct sample sub-groups. While caution is warranted, given the cross-sectional nature of our data and the likely existence of endogeneity between socio-economic status, oral hygiene habits and perceived treatment need, the comparison of results based on pooled analysis compared to those in which the sample is partitioned based on expressed treatment are stark in places. They clearly demonstrate the existence of distinct relationships across need, predisposing and enabling factors with respect, for example to predisposing factors. Thus, while being over 65 is associated with less recent and one might reasonably infer less frequent dental visits among those with perceived treatment need, among those without such needs, it is associated with more recent use. In the pooled analysis reported in Table 6 these distinct results are masked. Similar results are noted with respect to income with again a reversal of sign on the coefficients. That a clearer relationship between socio-economic status as reflected by income and service use is not evident contrasts with some other studies though care is warranted here in comparisons given differences in entitlements and access to dentists [30]. With respect to enabling factors such as the role of time off work again pooling data where different needs exist is seen to mask relationships that are apparent when the sub-groups are examined separately. The distinct impact of factors such as ability to find a dentist the respondent was comfortable with across groups differentiated by need is also stark.

Clearly the potential for erroneous policy advice to flow from pooled analyses exists. For example, with respect to enabling factors, the results suggest that addressing the issue of finding dentists the respondent feels comfortable with (a dentist they like) is more likely to yield dividends for individuals with perceived treatment need. For this group the opportunity cost of time may be less of an issue than the stigma associated with poor oral health, for example. By contrast, among those without perceived treatment needs, those for whom preventive services may be more sought after given their oral hygiene habits, the opportunity cost of time may be greater and addressing issues related to getting time off work may pay greater dividends. Given the lower prevalence of good oral hygiene habits among those with self-reported treatment need compared to those without, increased health education and promotion activities as part of a programme of secondary prevention targeted at those in receipt of restorative care seems warranted. More generally a one size fits all response to different needs among distinct sample sub-groups would not seem appropriate. While some previous studies that applied the Andersen model in the area of dentistry have sought to incorporate direct and indirect relationships between perceived treatment need, other factors and use of services [25] this has not always been the case [30]; our results suggest greater care is required when modelling need in dentistry.

Our study has a number of limitations. First, the data are cross sectional in nature and relationships should be interpreted as associations rather than necessarily being causal. Second, the data are self-reported and may be subject to reporting bias. Third, utilization is measured in terms of how recently the dentist was visited and as a categorical rather than continuous variable. While we have inferred frequency to be related to how recently the dentist was visited this assumption may not hold for all respondents.

## Conclusion

Disparities in oral health and service use related to socio-economic status have been observed in the United Kingdom. The Anderson behavioural model which delineates between predisposing, enabling and need factors has been used to examine the factors that influence the dental service use. In applying the Andersen model, it is important to take account of potential differences in the type of need individuals express, for example, in the case of dentistry the difference between restorative and preventive services when applying and interpreting the Andersen model. This study demonstrates the existence of distinct relationships between those with self-reported treatment needs and those without in terms of the interval since last dental visit.

## Supplementary Information

**Additional file 1.**

## Data Availability

The data that support the findings of this study are openly available in (UK Data Service) at https://beta.ukdataservice.ac.uk/datacatalogue/series/series?id=2000047. https://doi.org/10.5255/UKDA-SN-8737-1, reference number (8737).

## References

[1] Watt R, Sheiham A (1999). Inequalities in oral health: a review of the evidence and recommendations for action. Br Dent J.

[2] Locker D (2000). Deprivation and oral health: a review. Community Dent Oral Epidemiol.

[3] Sanders AE, Spencer AJ (2004). Social Inequality: Social inequality in perceived oral health among adults in Australia. Aust N Z J Public Health.

[4] Borrell LN, Baquero MC (2011). Self-rated general and oral health in New York City adults: assessing the effect of individual and neighborhood social factors. Community Dent Oral Epidemiol.

[5] Celeste RK, Nadanovsky P, de PonceLeon A, Fritzell J (2009). The individual and contextual pathways between oral health and income inequality in Brazilian adolescents and adults. Soc Sci Med.

[6] Turrell G, Sanders AE, Slade GD, Spencer AJ, Marcenes W (2007). The independent contribution of neighborhood disadvantage and individual-level socioeconomic position to self-reported oral health: a multilevel analysis. Community Dent Oral Epidemiol.

[7] Wamala S, Merlo J, Boström G (1978). Inequity in access to dental care services explains current socioeconomic disparities in oral health: the Swedish National Surveys of Public Health 2004–2005. J Epidemiol Community Health.

[8] Shelley D, Russell S, Parikh NS, Fahs M (2011). Ethnic disparities in self-reported oral health status and access to care among older adults in NYC. J Urban Health.

[9] Sabbah W, Tsakos G, Chandola T, Sheiham A, Watt RG (2007). Social gradients in oral and general health. J Dent Res.

[10] Borrell LN, Taylor GW, Borgnakke WS, Woolfolk MW, Nyquist L v (2004). Perception of general and oral health in White and African American adults: assessing the effect of neighborhood socioeconomic conditions1. Community Dent Oral Epidemiol.

[11] Morita I, Nakagaki H, Yoshii S, Tsuboi S, Hayashizaki J, Igo J (2007). Gradients in periodontal status in Japanese employed males. J Clin Periodontol.

[12] Elani HW, Harper S, Allison PJ, Bedos C, Kaufman JS (2012). Socio-economic inequalities and oral health in Canada and the United States. J Dent Res.

[13] Sanders AE, Spencer AJ (2005). Why do poor adults rate their oral health poorly?. Aust Dent J.

[14] Locker D (2009). Self-esteem and socioeconomic disparities in self-perceived oral health. J Public Health Dent.

[15] Sabbah W, Watt RG, Sheiham A, Tsakos G (2009). The role of cognitive ability in socio-economic inequalities in oral health. J Dent Res.

[16] Baskaradoss JK (2018). Relationship between oral health literacy and oral health status. BMC Oral Health.

[17] Steele J, O’ Sullivan I. Adult Dental Health Survey 2009: Executive Summary. https://files.digital.nhs.uk/publicationimport/pub01xxx/pub01086/adul-dent-heal-surv-summ-them-exec-2009-rep2.pdf. Accessed 23 June 2022.

[18] Shen J, Wildman J, Steele J (2013). Measuring and decomposing oral health inequalities in an UK population. Community Dent Oral Epidemiol.

[19] Guarnizo-Herreño CC, Watt RG, Fuller E, Steele JG, Shen J, Morris S (2014). Socioeconomic position and subjective oral health: Findings for the adult population in England, Wales and Northern Ireland. BMC Public Health.

[20] Donaldson AN, Everitt B, Newton T, Steele J, Sherriff M, Bower E (2008). The effects of social class and dental attendance on oral health. J Dent Res.

[21] Baker SR, Baker S (2009). Applying Andersen’s behavioural model to oral health: what are the contextual factors shaping perceived oral health outcomes?. Community Dent Oral Epidemiol.

[22] Watt RG (2012). Social determinants of oral health inequalities: implications for action. Community Dent Oral Epidemiol.

[23] Babitsch B, Gohl D, von Lengerke T. Re-revisiting Andersen’s Behavioral Model of Health Services Use: a systematic review of studies from 1998-2011. Psychosoc Med. 2012;9. 10.3205/psm000089 PMC3488807 23133505

[24] Andersen R, Newman JF. Societal and Individual Determinants of Medical Care Utilization in the United States. Milbank Q. 2005;83(4). 4198894

[25] Marshman Z, Porritt J, Dyer T, Wyborn C, Godson J, Baker S (2012). What influences the use of dental services by adults in the UK?. Community Dent Oral Epidemiol.

[26] Hajek A, Kretzler B, König HH (2021). Factors associated with dental service use based on the andersen model: a systematic review. Int J Environ Res Public Health.

[27] Cylus J, Richardson E, Findley L, Longley M, O’neill C, Steel D. Health Systems in Transition: United Kingdom Vol. 17 No. 5 2015. United Kingdom Health system review. 2015;17. https://www.euro.who.int/__data/assets/pdf_file/0006/302001/UK-HiT.pdf. Accessed 15 May 2022. 27049966

[28] Telford CJ, O’Neill C (2012). Changes in dental health investment across the adolescent years. British Dent J.

[29] Telford C, Murray L, Donaldson M, O’Neill C (2012). An analysis examining socio-economic variations in the provision of NHS general dental practitioner care under a fee for service contract among adolescents: Northern Ireland Longitudinal Study. Community Dent Oral Epidemiol.

[30] Galvão MHR, Medeiros A de A, Roncalli AG. Using Andersen’s behavioural model to examine individual and contextual factors associated with dental service utilization in Brazil. Community Dent Oral Epidemiol. 2022. 10.1111/CDOE.12753. 10.1111/cdoe.12753 35488515

