# Supplementary material for: A comparative examination of the role of need in the relationship between dental service use and socio-economic status across respondents with distinct needs using data from the Scottish Health Survey
Source: BMC Public Health. 2023 Jan 24;23:159. doi: 10.1186/s12889-023-15078-z (PMC9872289; doi:10.1186/s12889-023-15078-z)
Supplement: Supplementary file 1 — Additional file 1. [file 12889_2023_15078_MOESM1_ESM.docx]

***Appendix 1***

**) perceived treatment need)**

If you went to the dentist tomorrow, do you think you would need treatment?

1 Yes

0 No

**(Sex)**

ASK: Is (*name of respondent*) male or female?

1 Male

0 Female

**(Age)**

Can I check, what was *(name of respondent’s)* age last birthday?

Range: 0..120

1 over 65

0 65 and under

**(SmokeNow)**

Do you smoke cigarettes nowadays?

1 Yes

0 No

**(Service use)**

When visiting the dentist, do any of the following apply to you?

CODE ALL THAT APPLY

1 Difficulty in getting time off work [DentProb]

2 Difficulty in getting an appointment that suits me [DentPro2]

3 Dental treatment too expensive [DentPro3]

4 Long way to go to the dentist [DentPro4]

5 I have not found a dentist I like [DentPro5]

6 I cannot get dental treatment under the NHS [DentPro6]

7 I have difficulty in getting access, e.g. steps, wheelchair access [DentPro7]

8 Other [DentPro8]

**(DentHlth1)**

SHOW CARD K8

Which of the following do you do **daily** to improve your dental and oral health?

CODE ALL THAT APPLY.

INTERVIEWER: If respondent is unsure whether the toothpaste they use is fluoride or not, assume that it is and code 1.

1 Brush my teeth with fluoride toothpaste [DentHlt1]

2 Use dental floss [DentHlt2]

3 Use a mouth rinse [DentHlt3]

4 Restrict my intake of sugary foods and drinks [DentHlt4]

**(Degree)**

SHOW CARD Q1

Please look at this card and tell me which, if any, of the following educational qualifications you have.

CODE ALL THAT APPLY.

**None of these qualifications = Code 12**

1 School Leaving Certificate, National Qualification Access Unit [TopQua1]

2 O Grade, Standard Grade, GCSE, GCE O Level, CSE, National

Qualification Access 3 Cluster, Intermediate 1 or 2, National 4 or 5,

Senior Certificate or equivalent [TopQua2]

3 GNVQ/GSVQ Foundation or Intermediate, SVQ Level 1 or 2,

SCOTVEC/National Certificate Module, City and Guilds Craft,

RSA Diploma or equivalent [TopQua3]

4 Higher grade, Advanced Higher, CSYS, A level, AS Level, Advanced

Senior Certificate or equivalent [TopQua4]

5 GNVQ/GSVQ Advanced, SVQ Level 3, ONC, OND, SCOTVEC National

Diploma, City and Guilds Advanced Craft, RSA Advanced Diploma

or equivalent [TopQua5]

6 HNC, HND, SVQ Level 4, RSA Higher Diploma or equivalent [TopQua6]

7 First Degree, Higher degree, SVQ Level 5 or equivalent [TopQua7]

8 Professional qualifications e.g. teaching, accountancy [TopQua8]

9 Other school examinations not already mentioned [TopQua9]

10 Other post-school but pre Higher education examinations

not already mentioned [TopQua10]

11 Other Higher education qualifications not already mentioned [TopQua11]

12 No qualifications

Refused

Don't know

Schedule not applicable

Not applicable

Degree or higher

HNC/D or equivalent

Higher grade or equivalent

Standard grade or equivalent

Other school level

No qualifications
